# Pediatric Compounding Pharmacy: Taking on the Responsibility of Providing Quality Customized Prescriptions

**DOI:** 10.3390/children6050066

**Published:** 2019-05-04

**Authors:** Tricia Heitman, A. J. Day, August S. Bassani

**Affiliations:** PCCA, 9901 South Wilcrest Drive, Houston, TX 77099, USA; aday@pccarx.com (A.J.D.); gbassani@pccarx.com (A.S.B.)

**Keywords:** compounding, pediatric, allergies, unique needs, compounded, sensory processing disorder, compounding pharmacist, compounding pharmacy, complex needs, suspensions

## Abstract

Compounding pharmacy has an important role to play in the field of pediatric medicine. These specialized pharmacies can offer solutions to the unique patient needs that arise in the pediatric population. Medication can be tailored to the child to allow better compliance in cases when the commercial product is unable to meet the needs of the patient. For example, a suspension, suppository, or lozenge formulation is sometimes needed when the manufactured products are only offered as solid oral dosage forms. Sensory processing disorder (SPD), patients with food allergies, and specific dietary needs can also be a big challenge for caregivers and practitioners who need alternatives to the commercially available forms. Three example cases are presented to help describe the process of collaboration between the pharmacist, patient, and doctor to solve the patient’s needs.

## 1. Introduction

The United States Food and Drug Administration defines drug compounding as “the process of combining, mixing, or altering ingredients to create a medication tailored to the needs of an individual patient. Compounding includes the combining of two or more drugs” [1]. Compounding pharmacies exist because they fill unique patient needs. To make this happen, collaboration must take place between the practitioner, patient, and compounding pharmacist. Often, the needs of small patient groups, such as patients with rare disease states or allergies, may not be considered financially profitable by large pharmaceutical companies. This is especially true in the pediatric field of practice because of the variety of complex needs. In many cases, the practitioner will turn to compounding pharmacists who use pharmaceutical-grade ingredients to fill the prescription in their specialized, compounding lab. The practice of compounding pharmacy takes skill, training, and a desire to help others with their individual drug-related needs.

## 2. Medications Unavailable in an Appropriate Dosage Form

Sometimes a suspension, suppository, or lozenge formulation is needed when the manufactured products are only offered as solid oral dosage forms [2]. Most children aged 6–11 years can swallow a small oral tablet with training by a pharmacist or practitioner, but about 9% of children cannot [3]. Some common barriers to effective tablet swallowing include developmental stage [3], fear [4], anxiety, and intolerance to unpleasant flavors. Children with special healthcare needs (CSHCN) and/or medical complexity (CMC) will pose an even greater challenge, potentially risking the patient’s treatment outcome. A compounding pharmacist who is knowledgeable about the pharmaceutical chemistry of each drug can ensure that the patient receives a customized medication that is more likely to be tolerable. Factors such as pH, drug stability, and chemical compatibility are accounted for when deciding on the best delivery vehicle for the medication. Medications that are unstable in an aqueous environment will need a vehicle that is anhydrous, such as an oil or a flavored powder. Still, other medications that require enteric coating or slow-release kinetics may not be appropriate as a liquid. Collaboration with a compounding specialist can ensure that the delivery form is the right choice for both the patient and the prescribed medication.

Some medications commonly compounded in child-friendly dosage forms include losartan [5,6], atenolol [4,7], tizanidine [8], atomoxitine [9], sildenafil [10], mefloquin [11,12], metronidazole [13,14], and valacyclovir [11,15].

## 3. Sensory Processing Disorders

Sensory processing disorder (SPD) often occurs with developmental disorders, such as autism, fragile X syndrome, and attention-deficit hyperactivity disorder (ADHD). SPD is a neurological condition in which input from the senses is poorly detected, modulated, or interpreted, producing an abnormal response to stimuli from the environment [16]. The sensation of taste and texture in the mouth may trigger negative responses in children with this condition. The responses can include gagging, vomiting, and screaming, which will result in non-compliance [17]. Compounding pharmacists have a variety of dosage formulation options, such as capsules, tablets, solutions, suspensions, or even topical preparations. Sometimes modifying the recipe is simple, such as changing the color or using a familiar, favorite flavor to entice a child to accept a medication. Also, children with the sensory disorder tend to be sensitive to gritty textures. In these cases, the compounding pharmacist can reduce the particle size of the medications by grinding in a mortar and pestle so that the mouth feel is smoother for the patient. This is especially important for lozenges and suspensions. Choosing the right base can also create a better experience for the patient. A lozenge dosage form could be made in a gelatin, polyglycol, or fatty base, depending on the patient’s preference and needs. Suspensions or solutions can also be made in a variety of formulations that have a unique mouth-feel that appeal to the sensitive patient. In other cases, a topical dosage form is applied to the skin. Transdermal administration eliminates the need for oral delivery of the medication. In each case, the compounding pharmacist works collaboratively to help the patient comply with the prescribed treatment regimen. The compounding pharmacist can spend the time with the parents and child to offer choices and options for these children and caregivers when they feel they have none.

## 4. Food Allergies

Patients with food allergies can also be a big challenge for caregivers and practitioners. The foods that most often trigger childhood allergic reactions include eggs, cow’s milk, peanuts, tree nuts, soy, and wheat, and can affect up to 8% of children [18]. Compounding pharmacists are adept at meeting the needs of children with common and uncommon food allergies. Using pharmaceutical-grade ingredients and allergen-free bases, compounding pharmacists can provide a greater variety of medication options. Often, practitioners will turn to their compounding pharmacy to provide critical medications that would otherwise not be available. For example, high-fructose corn syrup and sorbitol, commonly used in commercial medications, must be avoided in children with corn allergies. A compounding pharmacist can choose a solution or suspension base and other ingredients that are free of these allergens.

## 5. Medically Prescribed Diets

Compounding pharmacists will often be asked to assist children with specific dietary needs. Two diets, the celiac diet and the ketogenic diet, are most commonly seen. Celiac disease can be seen in up to 3% of children under the age of 15 years [19,20]. Patients diagnosed with this condition will require a diet free of gluten. Gluten is found in many commercially available medications. It is difficult for patients sensitive to gluten to know if the medication contains gluten because the ingredient names are not always obvious [21]. Some of the hidden ingredients include dextrans, dextrins, pregelatinized starch, and sodium starch glycolate. Sometimes gluten-containing ingredients are used in the manufacturing process. The only true way to know is to contact the manufacturer of the medication. Compounding can help when a commercially available medication without gluten is unavailable. The ketogenic diet is another dietary intervention that is helpful in pediatric patients. This diet helps reduce seizure activity in patients who are refractory to antiepileptic medications by increasing ketone bodies from fat metabolism [22,23,24]. Patients prescribed with this low-carbohydrate and high-fat diet will need medications that have little or no carbohydrates. Adding medications that do not fit within the prescribed diet can sabotage the efforts of the practitioner and caregiver.

## 6. Clinical Compounding Specialists

When customizing medications, it is important to have a foundational knowledge base and strong research skills to seek unique solutions to challenging patient situations. Because the needs of our patients are unique and sometime rare, there may be a need for more exhaustive research of peer-reviewed literature than is possible with the time restraints of a busy medical office or pharmacy. For this reason, pharmacists and clinicians may seek input from a clinical compounding pharmacist with extensive training and compounding experience. The main objective of a clinical compounding pharmacist is to provide compounded drug information and specialized training programs for compounded medications. Clinical compounding pharmacists can help provide guidance in choosing the best dosage form, dosage, and formulation for specific patients. Clinical compounding specialists also provide assistance with calculations. Precise formulations are imperative to provide safe compounds to our patients. Example calculations include isotonicity and osmolarity calculations for sterile products, capsule formula calculations, adjustments for individual ingredient potency, suppository and lozenge calculations, and a variety of other critical computations. Collaborating with a specialist provides a resource with years of experience in direct patient care to empower the practitioner and pharmacist to provide a more patient-centered approach. Collaborative efforts can help close communication gaps and improve treatment outcomes by optimizing medication dosages and reducing adverse drug reactions. 

Example question categories taken by the clinical compounding specialists are as follows:

- Calculations;
- Dermatology;
- Dental;
- Ear, Nose, and Throat;
- Flavoring;
- Gastrointestinal;
- Hospice;
- Hormone Replacement Therapy;
- Men’s Health;
- Neurology;
- Pain;
- Pediatrics;
- Thyroid;
- Veterinary;
- Wound Care;
- Women’s Health.

Clinical compounding pharmacists are currently expanding their role to provide this service to members of the health-system community of care. Clinical specialists, training, and research would be available to the hospital pharmacy staff and practitioners. This would further expand the variety of compounded options and data available to those in inpatient and outpatient settings.

## 7. Example Cases

The parent of a male patient, 9 months old, presented to a compounding pharmacy with a prescription for valproic acid indicated for epilepsy. Although valproic acid is available commercially as a 250 mg/5 mL solution, the child was unable to utilize the solution because he was also on a medically prescribed ketogenic diet to help control his seizures [22,23,24]. The ketogenic diet requires calories high in fat, low in carbohydrates, and with an adequate amount of protein. Commercially available forms of valproic acid solution contain sorbitol, sucrose, and glycerin, which are high in carbohydrates. Therefore, the patient was sent to his local compounding pharmacy by his physician to have valproic acid compounded in an oral, carbohydrate-free solution. The pharmacy, after receiving the prescription, called a clinical compounding specialist for assistance with the formulation. The clinical compounding specialist researched formulation stability, bioavailability literature, and relevant regulatory parameters for the pharmacy. The clinical compounding specialist recommended a concentration of 50 mg/mL to be consistent with the commercially available solution and a solution base that would provide a carbohydrate-free dosage for the patient. The pure sodium valproate, European Pharmacopea (EP), was formulated into an aqueous solution utilizing steviol glycosides as a non-caloric, natural sweetener. The local compounding pharmacy compounded the solution for the patient who was treated successfully.

In another case, a pediatric neurologist contacted a local compounding pharmacist regarding a 7 year old female, who went from being very vibrant to non-responsive in the last year. The patient started having idiopathic seizures 12 months prior. In response, her physician tried several different traditional regimens, including valproic acid, gabapentin, and levetiracetam, which were later discontinued due to minimal improvement. The pediatric neurologist then ordered allergy testing [25] and tests for nutritional deficiencies. In response to the results, the neurologist prescribed a compounded levetiracetam, which excluded all flavors and artificial sweeteners [26,27,28]. The patient’s seizures started to improve immediately. The physician added other flavor- and artificial sweetener-free compounded nutritional supplements to the patient’s regimen, including alpha lipoic acid [23], coenzyme q10 [29], B vitamins [30], levocarnitine [31,32], and vitamins A, E, and D3. The clinical compounding specialist helped determine the best suspension vehicle for the supplement cocktail. After just one week, the patient started responding to her name and smiling in response to her mother. Her seizure frequency also started to decrease. After six months, the patient was able to sit unassisted and feed herself.

In a final example, an 8 year old male weighing 32 kg was prescribed malaria prophylaxis due to an impending stay in India. The family was planning an extended stay of three months with the child. Mefloquin [11,12] was prescribed at a dose of 160 mg. It was to be started two weeks prior to travel and finished four weeks after the family’s return from the endemic area. The child refused any solid dosage forms. Mefloquin, only commercially available as a 250 mg tablet, was unsuitable for the child. The family requested an oral suspension to take to India. Due to the long duration of therapy, beyond use date parameters for aqueous compounded dosage forms, and the poor aqueous solubility of the active ingredient, it was decided to compound a dry powder formulation to be dispensed with a suspending base, mixed at the time of use. It was decided that a combination of capsule and suspension mixed at the time of use would be the most suitable option. Compounded capsules allow compounders to provide a precise dosage and maintain the stability of the drug by keeping it in a dry environment. It is also not necessary to keep the capsules refrigerated, making travel much simpler. The local compounding pharmacy compounded the 160 mg mefloquin capsules with a microcrystalline filler utilizing a Jaansun^®^ capsule machine (PCCS, Houston, Texas, United States of America). A separate, aqueous suspension vehicle, Suspendit^®^ was given to cover the bitter taste of the drug. The compounding pharmacy added steviol glycosides as an added sweetener and a tutti-fruity flavor to Suspendit^®^ to aid in masking the bitterness. The child’s caregiver opened one capsule weekly and stirred it into the flavor-masking suspension using a dosage cup. The treatment was successful for the child and caregiver.

## 8. Conclusions

Compounded medications are an important piece of the patient care puzzle. For children with special healthcare needs and their caregivers, a compounding pharmacy offers them hope. When commercially available options are exhausted, a compounding pharmacist can help offer solutions to unique patient needs. Improved compliance, improved patient satisfaction, and reduced adverse reactions result when the correct compounding solution is utilized. By working together to provide customized, compounded medication, the result is unique solutions to patient needs that can improve the patient’s treatment outcome and elevate practitioner confidence.
